# Impact of Antibiotic Therapy with Ceftazidime Avibactam vs. Best Available Therapy in Adult Patients with Bacteremia Caused by Carbapenem-Resistant Enterobacterales

**DOI:** 10.3390/antibiotics14030226

**Published:** 2025-02-24

**Authors:** Daniel Arboleda, Camilo Buitrago, Erika Paola Vergara, Laura Cristina Nocua-Báez, Carlos Humberto Saavedra, Jorge Alberto Cortés

**Affiliations:** 1 Department of Medicine, Universidad Nacional de Colombia, Bogotá 111321, Colombia; darboledap@unal.edu.co (D.A.); cbuitragob@unal.edu.co (C.B.); evergarav@unal.edu.co (E.P.V.); lcnocuab@unal.edu.co (L.C.N.-B.); chsaavedrat@unal.edu.co (C.H.S.); 2 Infectious Diseases Service, Hospital Universitario Nacional, Bogotá 111321, Colombia; 3 Clínica Universitaria Colombia, Clínica Colsanitas, Bogotá 111321, Colombia

**Keywords:** Enterobacteriaceae infections, bacteremia, Klebsiella, carbapenemases, ceftazidime, drug resistance, bacterial

## Abstract

**Background/Objectives:** Carbapenem-resistant Enterobacterales (CRE) infection is associated with a higher mortality rate. The purpose of this study was to evaluate the effect of ceftazidime avibactam (CZA) for treating bacteremia caused by CRE compared to the best available therapy in an area where these microorganisms are endemic. **Methods**: A retrospective cohort study of patients with CRE bacteremia was conducted. We included adults with CRE bacteremia who were treated with CZA or the best available therapy (BAT) for more than 48 h, and the hospitalization time was recorded. The outcomes included death during hospitalization, relapse, and microbiological cure. Confounders were adjusted using propensity score-derived stabilized inverse probability of treatment weighting (IPTW). **Results**: A total of 169 patients with CRE bacteremia were included. About 72.6% of isolates had a class A serin carbapenamase, and 20.4% had metallo-β-lactamase co-production. A total of 107 patients were treated with CZA, 63% in monotherapy and 32% with aztreonam (ATM). Crude mortality during hospitalization was 36 (34.5%) in patients treated with CZA and 21 (33.2%) with BAT. No difference was observed between death rates (HR 0.86: IC 95% 0.40–1.83), microbiological cure (OR 1.31 IC 95% 0.46–3.67), clinical response (OR 1.39 IC 95% 0.35–5.43), acute kidney injury (OR 0.56 IC 95% 0.11–2.80) or relapse (OR 0.99 IC 95% 0.17–5.51) during the hospitalization after the adjustment. **Conclusions**: Among adult patients with CRE, no differences were observed between treatments with CZA and BAT after adjustment with IPTW.

## 1. Introduction

Carbapenem-resistant Enterobacterales represents a public health problem because of its great ability to spread, and it has become an endemic microorganism in many countries [1]. Infections caused by these bacteria are associated with a high mortality rate, ranging between 42% and 56% globally, as well as high health costs and morbidity [2]. This is due to the few available treatment options, frequent adverse reactions with these treatment options, and, inevitably, an increase in antimicrobial resistance [3,4,5].

In terms of new alternative treatments, some beta-lactamase inhibitors such as avibactam, vaborbactam, and relebactam have demonstrated promising outcomes in CRE infection mortality [5,6]. However, the available information remains limited, and there are no random clinical trials assessing these methods.

The aim of this study was to compare the mortality and other clinical outcomes of the treatment of CRE bacteremia with ceftazidime avibactam (CZA) and the best available therapy (BAT), defined as the most appropriate or best available treatment based on susceptibility or availability at the time other than CZA.

## 2. Results

**Figure 1:**
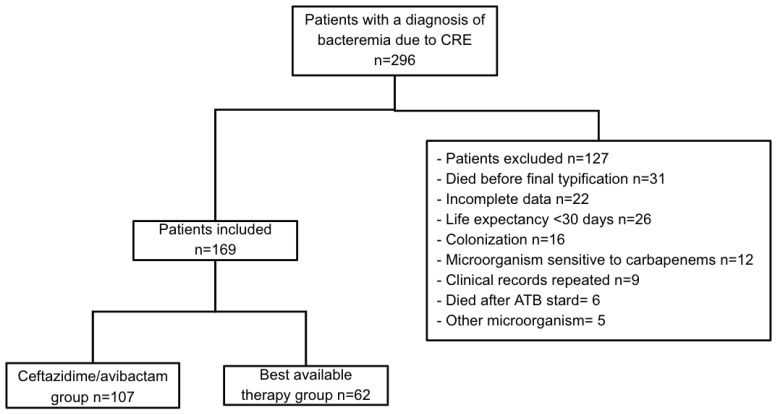
Patient cohort selection diagram.

A total of 296 medical records related to CRE bacteremia were registered in the system of antimicrobial resistance during the study time in the included institutions (Figure 1). A total of 169 medical records were included, of which 107 were from patients who had received CZA as treatment, while 62 were from patients who received BAT.

### 2.1. Sociodemographic Characteristics

Among the 169 included patients, 99 (58.6%) were men, and the average age was 55.3 (SD 16.64) years. The Charlson comorbidity mean index value was 4.1 (SD 2.7) and was almost the same in both groups (4.0 in the CZA group vs. 4.2 in the BAT group). A total of 30.8% of patients presented some form of surgical diagnosis, and this figure was greater in the BAT group (40.3%) than in the CZA group (25.2%).

The most frequent comorbidities were hematologic neoplasia, with 56 (33.1%) patients. The CZA group had more cases (41, 38.3%) than the BAT group (15, 24.2%). Secondary immunosuppression had a higher frequency in 59 (34.9%) patients: 40 (37.4%) in the CZA group and 19 (30.6%) in the BAT group (Table 1).

The mean SOFA score was 5.6 (5.8 CZA and 5.3 BAT), the mean INCREMENT score was 7.3 (7.4 CZA and 7.0 BAT) and the mean Pitt score was 2.1 (2.1 CZA and 2.1 BAT), and a similar frequency of septic shock and mechanical ventilation was found in the two groups (Table 1).

### 2.2. Microbiological Data

*Klebsiella pneumoniae* was found in 153 (90.5%) of all cases, *Serratia* spp. in 6 (3.6%), *Escherichia coli* in 4 (2.4%), *Citrobacter* spp. in 1 (0.6%), *Proteus* spp. in 1 (0.6%) and other less common Enterobacterales in 4 (2.4%). The infectious focus of bacteremia was secondary to intraabdominal infection in 43 (25.4%) and pneumonia in 36 (21.3%) cases and was associated with catheters in 33 (19%) patients. Primary bacteremia was present in 27 (16%) of the total cases, and the frequency was higher in the BAT group (22.6% vs. 17.8%). The remaining 30.3% of cases were related to infectious urinary tract disease in 21 (12.4%) and other foci in 9 (5.3%) cases.

Only 113 (66.8%) of all the cases had phenotypical screening or identification of the carbapenemase. Carbapenemase screening suggested that 82 (48.5%) cases involved serine-carbapenemase, 8 (4.7%) metallo-carbapenemase, and 23 (13.6%) a co-production. In patients who underwent immunochromatography, it was found that 28 (16.5%) of the cases involved *Klebsiella pneumoniae* carbapenemase (KPC), co-production of New Delhi metallo-β-lactamase (NDM) + KPC was recorded in 23 (13.6%) cases, NDM + Verona integron-encoded metallo-β-lactamase (VIM) in 1 (0.6%) case, and VIM in 1 (0.6%) case. There were differences in the distribution of the KPC cases; it was found in 24 (22.4%) of the patients that received CZA, but in only 4 (5.8%) of the BAT group (*p* = 0.003). Enzyme co-production was observed in 22 (26.8%) cases in the CZA group and 1 (3.2%) in the BAT group (*p* < 0.001). Non-D-class carbapenemase was obtained during the observation period.

### 2.3. Antimicrobial Therapy and Non-Adjusted Outcomes

Regarding the antibiotic therapy among the 169 patients, 107 (63.3%) received CZA, 68 (53.6%) in monotherapy, 35 (32.7%) with aztreonam (ATM), of which 28 had phenotypic confirmation, with 21 combinations of KPC plus metallo-β-lactamases and 4 (3.7%) in combination with another antibiotic, according to foci and susceptibility. Among 62 (36.7%) patients in the BAT group, 5 (8%) received monotherapy (four aminoglycosides and one tigecycline), and 43 (69.4%) received combined therapy, as shown in Table 2. The average length of antimicrobial therapy was 13.7 (11.3 SD) days in the CZA group vs. 12.5 in the BAT group, with 3.2 (2.9 SD) average days until the initiation of effective therapy adjustment, which was higher in the BAT group: 3.9 (3.4 SD) days vs. 2.8 (2.4 SD) in the CZA group. Twenty-three (13.6%) patients of the total (169) required another form of antimicrobial adjustment. In Table 3, the frequency of different non-adjusted outcomes is shown.

### 2.4. Survival Model and Adjusted Outcomes

**Figure 2:**
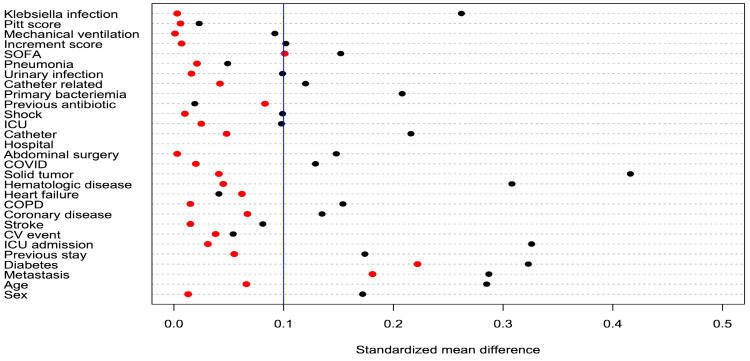
Standardized mean differences in unweighted (black points) and weighted samples (red points). ICU: Intensive care unit, COPD: Chronic obstructive pulmonary disease, CV: Cerebrovascular.

After weighting based on the inverse probability of treatment, a balance of most variables was achieved. The standardized differences between groups for all 29 covariables are seen in Table 1 and are compared before and after weighting in Figure 2.

**Figure 3:**
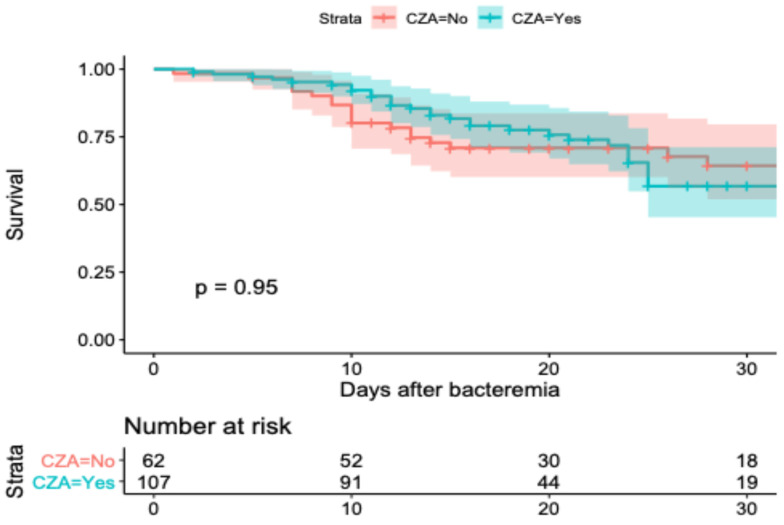
The 30-day Kaplan–Meier curves between patients with CZA treatment vs. BAT (CZA = No) using weighted data.

In Figure 3, the survival curve of weighted data is shown. In the Cox proportional risk model, an HR of 0.86 (IC 95% 0.40–1.83) for CZA use was identified, and an HR of 1.23 (IC 95% 0.52–2.91) for ATM was identified (Table 4).

Table 4 shows the adjusted outcomes after IPTW with different models. Hospital stay was evaluated for the surviving patients, and the survival curve showed a shorter length of stay for patients receiving CZA (*p* = 0.08); however, the Cox proportional model showed an HR of 1.30 (IC 95% 0.86–1.99) for CZA and an HR of 0.97 (IC 95% 0.56–1.66) for ATM.

### 2.5. Sensibility Analysis

In the multivariate logistic regression analysis of the mortality outcomes, it was found that the CZA-exposed group had an OR of 0.65 (IC 95% 0.26–1.62). The CZA + ATM combined group had an OR of 2.48 (IC 95% 0.86–7.12 *p* = 0.090). Other factors that were associated with the INCREMENT risk of mortality outcome are shown in Table 5.

## 3. Discussion

Among the 169-patient cohort of Colombian adults with carbapenem-resistant enterobacteria bacteremia and possible carbapenemase producers, we found that there was no statistically significant difference between in-hospital mortality in the CZA group and the BAT group in terms of clinical cure, microbiological cure, hospital stay, or infection relapse.

The most frequent enterobacteria was *Klebsiella pneumoniae*, and those that had a KPC enzymatic identification (72.6%) were the most commonly found, followed by KPC + co-expression of a metallo-β-lactamase, most frequently NDM. These findings are similar to previous studies in Colombia [7,8], which documented a high co-expression of metallo-β-lactamase enzymes (up to 13.6% of CRE cases), revealing the high dissemination of carbapenemase clones and metallo-β-lactamases in the country. The carbapenemase distribution found in this study in Colombia was similar to the data obtained from the United States and Europe [2,9,10].

In-hospital mortality in our study was lower than that reported by Zhou et al. (46.2%) [11], one of the largest cohorts available to date. However, in their study, the BAT group underwent a higher tigecycline scheme therapy in monotherapy or combined therapy (54%), in contrast to the BAT group in our study (15.5%). This can explain the lower mortality observed because many publications have documented a higher mortality in tigecycline-scheme-based therapy. In Tasin E et al.’s meta-analysis of 14 randomized trials with a total of 7400 patients, tigecycline use was associated with a higher mortality rate in some scenarios, especially unrelated intraabdominal or skin and soft tissue infections [12]. Satlin MJ et al. found a mortality rate similar to that of our study (38%), with intraabdominal infections being the predominant foci of bacteremia (33%) and 10% mortality in the CZA group vs. 31% in the BAT group based on polymyxin, without statistically significant differences [13]. These findings were consistent with the findings of our study, which reports a mortality of 34.5% in the CZA group vs. 33.2% in the BAT group, with no statistically significant differences. Another study conducted by Falcone et al. with a cohort of 102 patients with bacteremia reported a 19.2% mortality in the CZA group + ATM vs. 44% in the BAT group (*p* = 0.007). The principal infection in the Falcone et al. study was urinary infection (33, 32.4%) [14], whereas in our study, this etiology was less frequent. Furthermore, Falcone et al. described a minor proportion of patients in septic shock (27, 26.5%) with a median SOFA score of 4 points, which is lower than that reported in our study (5.6) [14].

In our study, those patients with urinary infection as the bacteremia focus had a more benign outcome OR of 0.08 (IC 95% 0.008–0.91), similar to the results reported by Vincent HT et al. and So-Ngern A. et al., in which the urinary infection focus of bacteremia had OR values of 0.194 (IC 95% 0.024–1.54) [15] and 0.16 (IC 95% 0.61–0.44), respectively [16].

Regarding the use of CZA monotherapy for the mortality outcome, our findings were congruous with those previously reported by Zheng G et al. in their cohort retrospective study of 164 patients. The comparison of the use of CZA vs. polymyxin in critical patients with CRE infection shows an HR of 0.591 (IC 95% 0.332–1.054) for this outcome, with statistical non-significance [17]. This evidence shows that the study population in different scenarios was heterogeneous and had different risk factors, treatment schemes, and distinct outcomes, which made it more difficult to compare our results to those published in the literature to date. Clearly, mortality is lower than in other scenarios, and the use of effective antimicrobial schemes is higher, explaining why there are no differences between both groups. In Chen J et al.’s meta-analysis, 4 of the 10 identified studies showed no differences in mortality between the CZA group and the BAT one based on polymyxin [18].

Our study has many limitations. As this was a retrospective observational study without any treatment randomization, bias could have affected the results. For this reason, we employed an IPWT model to balance the weight of the distinct variables that contributed to the outcomes. The use of the IPTW model allowed us to balance the differences among the variables included. An important variable that was not balanced was the previous history of diabetes, which has not been seen as an independent predictor of mortality in this group of patients [19].

Another limitation was that there were no data on the phenotype (i.e., the specific expression of resistance and certain enzymes) of all the isolates. We also did not identify a CZA MIC to evaluate the real percentage of resistance. Some patients may have received inappropriate treatment for potentially present enzymes. Data from Latin America shows that resistance to CZA is related to the presence of MBL, specifically the identification of NDM genes, either alone or in combination with KPC [20]. The presence of potential MBL explains the use of ATM in addition to CZA observed in approximately one-third of the patients. Current laboratory evidence has shown a potential beneficial effect of this combination [21], while the clinical data are limited and with moderate or serious risks of bias. However, these data suggest a benefit of the combined use of ATM and CZA [22]. In this study, the use of ATM did not affect the selected outcomes, but the sample size and the lack of complete information on the enzymes produced prevented us from establishing a relationship. Further studies are therefore required, in particular, randomized clinical trials.

A strength was that the analysis used in this study achieved a balance for most of the identified variables as possible outcome distractors. It is also remarkable that a greater number of patients were studied compared to most of the available small-cohort studies that evaluate CTZ treatment [17]. The sensibility analysis was also congruent with the findings identified in the primary analysis using IPTW, with closely related results.

## 4. Materials and Methods

### 4.1. Study Design and Population

This study involved a retrospective cohort in two third-level centers, Hospital Universitario Nacional de Colombia and Clinica Universitaria Colombia, in Bogota, Colombia, an endemic area for Enterobacterales microorganisms. These centers are referenced in clinical and surgical etiologies, including oncology, transplant, and critical care services. Adult patients (≥18 years of age) hospitalized between 1 January 2018 and 31 June 2023 who had CRE bacteremia treated with CZA or BAT for more than 48 h were included. The exclusion criteria were as follows: patients with anticipated do-not-resuscitate orders or with a life expectancy of no longer than 30 days; patients with incomplete medical records; and patients for whom there was no chance for a clinical follow-up, e.g., because of transfer to another hospital.

### 4.2. Study Participants

Prior to medical prescription for a bacteremia diagnosis, two exposure groups were defined as mutually exclusive. Those who initially received the antibiotic CZA or who were treated with CZA after the definitive identification of the bacteria were placed in the CZA group. Any other antibiotic treatments were considered part of the BAT group (including but not limited to beta-lactamas, tygecicline, aminoglycosides, and polimyxins).

### 4.3. Endpoints

The primary endpoint was mortality during hospitalization after a bacteremia diagnosis. Secondary endpoints were infection relapse, clinical response, microbiological cure, and acute kidney injury. An infection relapse was defined as a second infection caused by the same microorganisms that were previously isolated, even in patients whose therapy was suspended because of medical improvement that did not have a negative control culture. A patient was considered to have a favorable clinical response when none of the following criteria were present: death before day 21, persistent infection signs or symptoms at 21 days (about 3 weeks) of therapy with CZA or BAT or infection relapse. A microbiological cure was defined as the absence of the initial pathogen in the control cultures that were taken at the infection site on the fifth day of treatment. Mortality attributed to the infection was considered when the dissemination of infectious disease was implicated in multiorgan failure or septic shock and death. The definitive antibiotic time was considered as follows: days between the taking of blood cultures and the beginning of definitive antibiotic treatment (either CZA or BAT). Acute kidney injury was defined as a 1.5-fold increase in initial creatinine during the first 7 days after the initiation of antibiotic therapy.

### 4.4. Definitions and Covariations

Possible confounding variables were identified by addressing mortality predictors known from the literature [23,24]. The following features were considered: sociodemographic data (age and sex), comorbidities (heart failure, COPD, diabetes and oncologic neoplasia), microbiological factors (identification of *K. pneumoniae* and bacteremia foci), severity predictors of the disease (Pitt bacteremia score, INCREMENT mortality score and SOFA score) [19], admission to an ICU or previous admission to this service, development of septic shock and invasive mechanical ventilation (IMV). INCREMENT, SOFA, and Pitt scores were calculated for the development date of bacteremia.

### 4.5. Microbiology

Microbiological isolates were analyzed in a clinical laboratory using VITEK systems (Biomerieux, Marcy-l’Étoile, France) in Clinica Universitaria Colombia and Phoenix (Middletown, PA, USA) in Hospital Universitario Nacional for identification and susceptibility testing. The isolates that exhibited minimal inhibitory concentrations ≥4 mcg/mL for meropenem, according to the cut-off of the Clinical & Laboratory Standards Institute (CLSI) [25], were considered resistant to carbapenems and were sent for confirmation. Enzyme identification was screened with the use of phenylboronic and EDTA-based tests that test for the presence of serine (KPC) enzymes and MBL [26], respectively. Identification of the current enzymes of the isolates was achieved with an immunochromatographic assay, NG-Test Carba 5 (NG-Biotech, Messac, France), based on the availability of the test.

### 4.6. Statistical Analysis

The median standard deviation or median and interquartile range were used according to the distribution of the variable for the continuous variables and frequencies and rates for categorical variables. The primary analysis was performed using a propensity score to determine similar risk groups based on the used covariables; to establish the propensity score, a multivariate logistic regression model was established for another endpoint, CZA exposition. Adjusted analyses were performed using the inverse probability of weight based on exposition variables, and stabilized weights were assigned. The evaluation of the balance was conducted through graphical methods that compare the distribution of continuous baseline covariates between treated and control subjects and by using the weighted standardized median difference (SMD) to compare means, prevalences, higher-order moments, and interactions. Groups with an SMD of 0.1 or less were interpreted as balanced [27].

Afterward, a pseudo-population was created that was used to evaluate mortality through a survival model (Cox risk proportional model), where the outcome variable (dependent) was death time, and the predictable variable was CZA therapy, taking into account that the proportionality assumptions were met. Some patients underwent ATM therapy, so this was included as a new variable in the mortality models. Comparison was performed using the χ^2^ test for proportional comparison and survival curves (via the Log rank test), bearing in mind a threshold of *p* < 0.05 for statistically significant results. Dichotomic outcomes (clinical cure, relapse, and renal failure) were used in a multivariable logistic regression model, including the exposition variable. All these models used robust errors to calculate the confidence interval. Statistical analyses were conducted in R software (ver. 4.0.2, Vienna, Austria).

### 4.7. Ethics

The ethics committees of the participating centers approved the study (CEI-HUN-ACTA-2022-10, 4 August 2022 and CEI CUC-ACTA 045-23, 13 June 2023). Good practice in investigating recommendations was followed, information remained confidential, and an exception to informed consent was claimed because the study design was retrospective and did not represent any risk to the participants.

## 5. Conclusions

Our study shows that there are non-statistically significant differences in mortality between treatments with CZA and BAT. The mortality rate was high in the patient group treated with CZA; however, the early stages of effective antibiotic treatment can impact mortality. The selection of treatment does not affect secondary outcomes such as microbiological cure, clinical response, relapse, or hospital stay and is not related to acute kidney injury.

## Figures and Tables

**Table 1:** Sociodemographic characteristics of patients before and after analysis with reverse probability.

	Original Cohort (N = 169)			Post-IPWT		
	CZA (n = 107)	BAT (n = 62)	SMD	CZA	BAT	SMD
Male sex, n (%)	66 (61.7)	33 (53.2)	0.172	62.3 (59.6)	36.8 (58.9)	0.013
Age, years; mean (SD)	53.5 (16.5)	58.2 (16.7)	0.285	54.7 (16.5)	53.6 (17.2)	0.066
Charlson score, mean (SD)	4.0 (2.5)	4.2 (3.0)	0.057	3.9 (2.4)	3.7 (2.9)	0.083
Comorbidities, n (%)						
Acute heart attack	15 (14.0)	6 (9.7)	0.135	13.3 (12.7)	6.6 (10.5)	0.067
COPD	6 (5.6)	6 (9.7)	0.154	7.2 (6.9)	4.5 (7.2)	0.015
Diabetes mellitus	25 (23.4)	7 (11.3)	0.323	19.2 (18.3)	6.6 (10.6)	0.222
Chronic kidney failure	23 (21.5)	8 (12.9)	0.229	19.1 (18.3)	7.0 (11.3)	0.199
Heart failure	19 (17.8)	12 (19.4)	0.041	17.6 (16.8)	9.1 (14.6)	0.062
Hematology tumor	41 (38.3)	15 (24.2)	0.308	35.6 (34.0)	22.5 (36.1)	0.045
Solid tumor	16 (15.0)	20 (32.3)	0.416	22.5 (21.5)	14.5 (23.2)	0.041
COVID-19	14 (13.1)	11 (17.7)	0.129	15.8 (15.1)	9.0 (14.4)	0.020
Immunosuppression	40 (37.4)	19 (30.6)	0.143	36.1 (34.5)	28.3 (45.4)	0.224
Abdominal surgery	21 (19.6)	16 (25.8)	0.148	22.8 (21.8)	13.7 (22.0)	0.003
Surgical disease	27 (25.2)	25 (40.3)	0.326	30.4 (29.1)	17.3 (27.7)	0.031
Previous CRE infection	8 (7.5)	3 (4.8)	0.098	8.3 (7.9)	8.9 (14.2)	0.202
Previous antibiotic treatment	82 (76.6)	47 (75.8)	0.019	81.8 (78.2)	50.8 (81.5)	0.083
Previous hospital stay in days, mean (SD)	17.7 (16.8)	21.5 (25.4)	0.174	17.8 (17.7)	18.8 (20.6)	0.055
Urinary catheter	7 (6.5)	8 (12.9)	0.216	9.2 (8.8)	6.4 (10.2)	0.048
Severity of the disease, n (%)						
Critical care stay	57 (53.3)	31 (48.4)	0.098	53.5 (51.2)	31.2 (50.0)	0.025
Septic shock	38 (35.5)	25 (40.3)	0.099	38.2 (36.6)	23.1 (37.0)	0.010
SOFA, mean (SD)	5.8 (3.1)	5.3 (3.4)	0.152	5.6 (3.14)	5.3 (3.56)	0.101
INCREMENT, mean (SD)	7.4 (3.8)	7.02 (3.9)	0.102	7.3 (3.8)	7.3 (3.9)	0.007
Pitt score, mean (SD)	2.1 (2.5)	2.1 (2.6)	0.023	2.1 (2.5)	2.1 (2.6)	0.006
Invasive mechanical ventilation	35 (32.7)	23 (37.1)	0.092	36.0 (34.4)	21.4 (34.3)	0.001

BAT: best available treatment; Post-IPTW: Data from weighted sample; SMD: standardized mean difference; SD: standard deviation; COPD: chronic obstructive pulmonary disease; CRE: carbapenem-resistant Enterobacterales.

**Table 2:** Antimicrobial treatment in best available therapy (BAT) group.

Treatment Option	N (%)
Monotherapy	5 (7.2)
Combined antimicrobial therapy	57 (82.6)
Based on carbapenems	42 (60.9)
Doble carbapenem	14 (20.3)
Based on colistin/polymyxin	24 (34.8)
Tigecycline use	16 (23.2)
Aminoglycoside use	9 (13.0)
Quinolone use	8 (11.6)
Fosfomycin use	6 (8.6)

**Table 3:** Crude outcomes in original cohort, according to group.

	Total N = 169	CAZ/AVI (n = 107)	BAT (n = 62)
Length of hospitalization, days (SD)	41.5 (27.8)	40.9 (26.8)	43.7 (26.2)
Length of antibiotic therapy, days (SD)	13.7 (11.3)	13.3 (10.8)	12.9 (9.8)
Length of post-antibiotic treatment, days (SD)	22.4 (17.2)	21.3 (16.4)	21.07 (15.1)
Acute kidney injury, n (%)	17 (10.0)	7.0 (6.6)	10.0 (15.4)
In-hospital mortality, n (%)	57 (34.0)	36 (34.5)	21 (33.2)
In-hospital death caused by the infection, n (%)	44 (76.4)	25 (68.9)	19 (90.7)
Infection relapse, n (%)	21 (13.0)	14 (12.8)	7 (10.5)
Clinical response, n (%)	106 (62.7)	66 (61.8)	40 (61.6)
Microbiological cure			
Yes, n (%)	14 (8.3)	6.0 (5.6)	8.0 (12.3)
No, n (%)	24 (14.4)	22 (20.6)	2 (2.8)
Not applicable	131 (77.3)	78 (73.8)	53 (48.9)

SD: Standard deviation.

**Table 4:** Adjusted outcomes for treatment with CZA vs. BAT in adjusted cohort.

	Measure of Association	Value	Lower Limit	Upper Limit
CZA in-hospital mortality	HR	0.86	0.40	1.83
ATM in-hospital mortality	HR	1.23	0.52	2.91
Microbiological cure	OR	1.31	0.46	3.67
Clinical response	OR	1.39	0.35	5.43
Acute kidney injury	OR	0.56	0.11	2.80
Relapse	OR	0.99	0.17	5.51

CZA: Ceftazidime/avibactam, BAT: Best available treatment, ATM: aztreonam, HR: Hazard ratio, OR: Odds ratio.

**Table 5:** Multivariate analysis of associated mortality of all patients.

Variable	OR	Lower Limit	Upper Limit	*p*-Value
Ceftazidime/avibactam use	0.65	0.26	1.62	0.362
Aztreonam use	2.48	0.86	7.12	0.090
Charlson score	1.27	1.08	1.50	0.004
Urinary infection	0.08	0.008	0.91	0.042
SOFA	1.55	1.31	1.84	0.000
Previous stay before bacteremia	1.02	1.00	1.04	0.003
Bacteremia time before definitive antibiotic therapy (per day)	1.15	1.00	1.33	0.042

SOFA: SOFA score.

## Data Availability

The raw data supporting the conclusions of this article will be made available by the authors on request.

## Abbreviations

The following abbreviations are used in this manuscript:

CRE	Carbapenem-resistant Enterobacterales
CZA	Ceftazidime/avibactam
BAT	Best available therapy
ATM	Aztreonam
SMD	Standardized mean difference
SD	Standard deviation
